# Racial Differences in Post-Acute Transition After Hip Fracture in Medicare Patients With ADRD

**DOI:** 10.1093/geroni/igaa057.2307

**Published:** 2020-12-16

**Authors:** Indrakshi Roy, Amol Karmarkar, Amit Kumar, Meghan Warren, Patricia Pohl, Stefany Shaibi, Maricruz Rivera-Hernandez, James Rudolph

**Affiliations:** 1 Northern Arizona University, flagstaff, Arizona, United States; 2 Virginia Commonwealth University Health, Richmond, Virginia, United States; 3 Northern Arizona University, Flagstaff, Arizona, United States; 4 Northern Arizona University, Phoenix, Arizona, United States; 5 Brown University School of Public Health, Providence, Rhode Island, United States; 6 U.S. Department of Veterans Affairs Medical Center, Providence, Rhode Island, United States

## Abstract

The incidence of hip fractures in patients with Alzheimer’s disease and related dementias (ADRD) is 2.7 times higher than it is in those without ADRD. However, there are no standardized post-acute transition models for patients with ADRD after hip fracture. Additionally, there is a lack of knowledge on how post-acute transitions vary by race/ethnicity. Using 100% Medicare data (2016-2017) for 120,179 older adults with ADRD, we conduct multinomial logistic regression, to examine the association between race and post-acute discharge locations (proportion discharged to skilled nursing facility [SNF], inpatient rehabilitation facility [IRF], and Home with Home Health Care [HHC]), after accounting for patient characteristics. Compared to non-Hispanic Whites, Hispanics have a significantly lower odds ratio for discharge to HHC 0.62 (95%CI=0.53-0.73), IRF 0.44 (CI=0.39-0.51), and SNF 0.26 (CI=0.23-0.30). Improving care in patients with ADRD and reducing racial and ethnic disparities in quality of care and health outcomes will be discussed.

